# *Spiradiclis pengshuiensis* (Ophiorrhizeae, Rubioideae), a new species from Chongqing, China

**DOI:** 10.3897/phytokeys.63.8016

**Published:** 2016-05-18

**Authors:** Bo Pan, Hu-Sheng Ma, Rui-Jiang Wang

**Affiliations:** 1 Guangxi Institute of Botany, Guangxi Zhuangzu Autonomous Region and Chinese Academy of Sciences, Guilin 541006, China; 2 Key Laboratory of Plant Resources Conservation and Sustainable Utilization, Guangdong Provincial Key Laboratory of Applied Botany, South China Botanical Garden, Chinese Academy of Sciences, Guangzhou 510650, China

**Keywords:** China, New taxon, Rubiaceae, Spiradiclis

## Abstract

*Spiradiclis pengshuiensis* Bo Pan & R. J. Wang (Rubiaceae) is described as a new species from Chongqing in SW China. It is morphologically compared with *Spiradiclis pauciflora* L. Wu & Q. R. Liu because of their similarities in habit, pubescent surface, small leaf laminas and subglobose capsules. Its conservation status is evaluated as “VU” according to the IUCN categories and criteria.

## Introduction

The genus *Spiradiclis* Blume (Rubiaceae) mainly distributed in the tropical and subtropical limestone areas of Southern China and Northern Vietnam. Geographically, the localities of *Spiradiclis* species can extend northward to Mt. Emei (29°33'N) in Sichuan province of China and southward to Central Java (ca. 7°10'S) of Indonesia. The genus comprise about 50 species and ca. 91% species can be found in China, the center of species diversity (Chen and Taylor 2011, Deng et al. 2014, Wang et al. 2015, Wen et al. 2015, Wu et al. 2015, Wang 2016). *Spiradiclis* species are usually similar to *Ophiorrhiza* L. because of their similar inflorescence and flower characters, but the former is characterized by globose or ovoid (vs. strongly laterally compressed in *Ophiorrhiza*) capsules.

During a botanical inventory in Pengshui County, the east of Chongqing Municipality, in 2013, the senior author found a striking *Spiradiclis* species growing on the dry cliffs. In order to get enough materials for morphological observation and comparison, we successively collected the vouchers during the flowering and fruiting seasons in recent years. This new species can be readily distinguished from other *Spiradiclis* species in habit and flowers and herein described and illustrated.

## Material and methods

All materials were collected by ourselves and deposited at the herbarium of South China Botanical Garden, Chinese Academy of Sciences
(IBSC). The morphological data were collected by a Digimatic Caliper (Mitutoyo, Japan).

## Taxonomy

### Spiradiclis pengshuiensis

Taxon classificationPlantaeGentianalesRubiaceae

B. Pan & R. J. Wang sp. nov.

urn:lsid:ipni.org:names:77154905-1

Figure 1

#### Diagnosis.

*Spiradiclis pengshuiensis* is similar to *Spiradiclis pauciflora* L. Wu & Q. R. Liu, from which it differs in having linear (vs. triangular) stipules, 4–6 (vs. 3–4) secondary veins each side of the leaf laminas, salverform (vs. funnelform) corolla with 9–15 mm (vs. 7–9 mm) long tubes (Table 1).

**Table 1.** Morphological comparison of *Spiradiclis pengshuiensis* and *Spiradiclis pauciflora*.

Characters	*Spiradiclis pengshuiensis*	*Spiradiclis pauciflora*
Habit	Erect or prostrate at base	Creeping or with upper parts ascending
Indument	Pubescent whole plant surfaces	Pubescent whole plant surfaces
Stem	Terete	Terete
Stipules	Linear, 0.9–1.2 mm long	Triangular, less than 1 mm
Petioles length (mm)	3.5–23.5	3–10
Leaf blades	Ovate; base cuneate or late cuneate, asymmetry, decurrent; apex obtuse to rounded	Ovate to elliptic-ovate; base obtuse to broadly cuneate; apex obtuse to acute
Leaf texture	Papery	Papery
Leaf size (mm)	(4–) 7–15(–25) × (2.5–) 5–10 (–15)	5–20 × 5–15
Leaf color	Green adaxially and pale green abaxially	Olive-green adaxially, pale or sometime purplish red abaxially
Secondary veins on each side	4–6	3–4
Inflorescence	Terminal, 1–7(–12)-flowered	Terminal, 3–7(–9)-flowered
Peduncle length (cm)	1–2.1	1–3
Pedicel length (mm)	0–2	0.3–3
Calyx lobes	Lanceolate, 0.8–1.5 mm long	Ovate-triangular, 1.2–1.6 mm long
Corolla	Salverform; corolla tube 9–15 cm long; lobes oval, 5–7.5 mm long	Funnelform; corolla tube 7–9 mm long; lobes ovate-triangular, 2.5–3 mm long
Pin flowers	Corolla pubescent adaxially, without ring hairs; styles 9–12 mm long	Ring hairs present in adaxial side of corolla; styles 7.5–8.5 mm long
Style length in thrum flowers (mm)	ca. 5	2.7–3
Capsules	Subglobose, 3–5 mm in diam.	Subglobose, ca. 2 mm in diam.
Phenology	Flowering in November to next January, fruiting in December to February	Flowering in March to June, fruiting in May to August

#### Type.

CHINA. Chongqing Municipality: Pengshui County, Hanxia Town, Baixiang Village, 29°8'N, 108°6'E, 360 m alt., 29 Nov 2014, Rui-Jiang Wang 2931, long-styled flower (holotype IBSC; isotypes IBSC).

#### Description.

Perennial herbs, 4–9 cm tall, densely pubescent on plant surfaces; stems erect or prostrate at base and then ascending, terete, rooting at nodes; inter nodes 2–15 mm long. Stipules linear, 0.9–1.2 mm long. Petiole (3.5–)7–12(–23.5) mm long. Leaf blades opposite, ovate, (4–)7–15(–25) × (2.5–)5–10(–15) mm; base cuneate or late cuneate, asymmetry, decurrent; apex obtuse to rounded, papery, green adaxially and pale green abaxially; secondary veins 4–6 on each side; margin revolute slightly. Inflorescence terminal, cymose, 1–7(–12)-flowered but only 1–3 blooming simultaneously; peduncles 1–2.1 cm long; bracts and bracteoles linear, 1–2 mm long. Flowers distylous; pedicels 0–2 mm long; hypanthium obconical, 1–2 mm long; calyx lobes 5, lanceolate, 0.8–1.5 × ca. 0.4 mm. Corolla salverform, white adaxially, pinkish to white abaxially, pubescent both sides; tube 9–15 × 1.5–2 mm; lobes (4–)5, oval, 5–7.5 × 2.5–3.5 mm. Stamens 5; anthers linear-oblong, 1.5–2 mm long, dorsi-fixed. Stigma bilobed, lobes ovoid; ovary 2-celled, ovules many, axile. Long-styled flowers: stamens included; filaments adnate to the lower corolla tube, ca. 1.5 mm long; styles 9–12 mm long; stigma ca. 0.5 mm long, red to brown, extended to corolla throat, not exserted. Short-styled flowers: stamens extended to corolla throat, not exserted; filaments adnate to upper corolla tube, ca. 0.5 mm long; styles ca. 5 mm long; stigmas ca. 1.5 mm long, included. Capsules subglobose, 3–5 mm in diam., with persistent calyx lobes, dehiscent septicidally and loculicidally; valves 4, ovate, 3–5 mm long. Seeds 15–20 per capsule, ca. 0.5 mm long, brown, irregular pyramid to cuboid, foveolate on surface.

**Figure 1.**
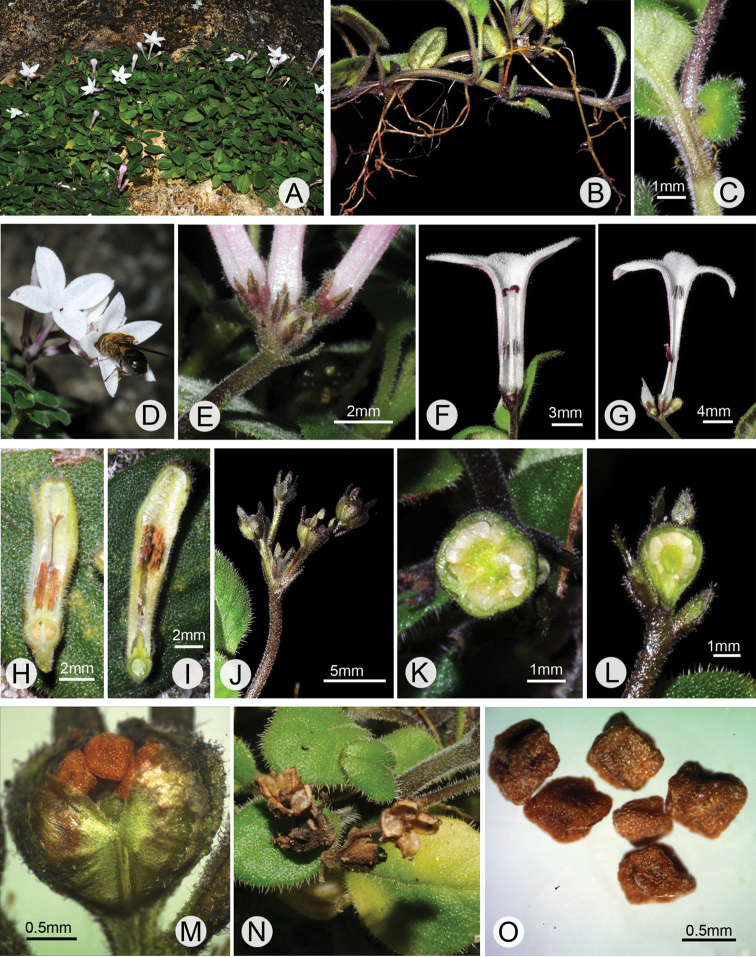
*Spiradiclis pengshuiensis* sp. nov. **A** habitat **B** habit **C** linear stipules **D** visiting insects **E** bracts and hypanthium **F–I** longitudinal section of long- (**F, H**) and short-styled flowers (**G, I**), respectively, showing the induments, relative positions and morphology of the stigmas and anthers and the developing capsules in distylous flowers **J** infructescence **K–L** transverse and longitudinal section of young capsules, respectively **M** mature capsule **N** dehiscent capsules **O** seeds. Photos by Ruijiang Wang.

#### Phenology.

Flowers from November to next January; fruits from December to next February.

#### Distribution and habitat.

*Spiradiclis pengshuiensis* is known only from the type locality. Its habitat is on the cliffs nearby the A-Yi-He river but with very poor conditions.

#### Conservation status.

Only three small populations and less than 500 individuals of *Spiradiclis pengshuiensis* within 5 km^2^ were found in the A-Yi-He Scenic Area. The tourist activity is the primary factor in the decline of populations. In addition, the plants always grow on the cliffs with little soil and insufficient water, which limited the development and dispersal of the species. Therefore we assign a preliminary IUCN threat status of Vulnerable [VU, B2ab(ii, iii, iv)] to *Spiradiclis pengshuiensis* (IUCN 2001).

#### Specimens examined.

**CHINA. Chongqing Municipality**: Pengshui County, Hanxia Town, Baixiang Village, 29°8'N, 108°6'E, 350 m alt., 29 Nov 2014, short-styled flowers, Rui-Jiang Wang 2932, 2937 (IBSC); same locality, 12 Jan 2016, fruiting, Rui-Jiang Wang 3095, 3099 (IBSC).

## Supplementary Material

XML Treatment for Spiradiclis pengshuiensis
